# Implementation outcomes of evidence-based quality improvement for depression in VA community based outpatient clinics

**DOI:** 10.1186/1748-5908-7-30

**Published:** 2012-04-11

**Authors:** John Fortney, Mark Enderle, Skye McDougall, Jeff Clothier, Jay Otero, Lisa Altman, Geoff Curran

**Affiliations:** 1 Health Services Research and Development, Central Arkansas Veterans Healthcare System, North Little Rock, AR, USA; 2 South Central Mental Illness Research Education and Clinical Center, Central Arkansas Veterans Healthcare System, North Little Rock, AR, USA; 3 Department of Psychiatry, University of Arkansas for Medical Sciences, Little Rock, AR, USA; 4 Office of Quality and Safety, Department of Veterans Affairs, Washington, DC, USA; 5 Desert Pacific Healthcare Network (VISN 22), Long Beach, CA, USA; 6 Behavioral Medicine Service, VA Loma Linda Healthcare System, Loma Linda, CA, USA; 7 Primary/Ambulatory Care, Greater Los Angeles Healthcare System, Los Angeles, CA, USA; 8 Department of Medicine, University of California, Los Angeles, CA, USA; 9 At the time the study was conducted, Dr. Enderle was affiliated with the South Central Veterans Healthcare Network (VISN 16; 10 At the time the study was conducted, Dr. Clothier was affiliated with the Mental Health Service, Central Arkansas Healthcare System

## Abstract

**Background:**

Collaborative-care management is an evidence-based practice for improving depression outcomes in primary care. The Department of Veterans Affairs (VA) has mandated the implementation of collaborative-care management in its satellite clinics, known as Community Based Outpatient Clinics (CBOCs). However, the organizational characteristics of CBOCs present added challenges to implementation. The objective of this study was to evaluate the effectiveness of evidence-based quality improvement (EBQI) as a strategy to facilitate the adoption of collaborative-care management in CBOCs.

**Methods:**

This nonrandomized, small-scale, multisite evaluation of EBQI was conducted at three VA Medical Centers and 11 of their affiliated CBOCs. The Plan phase of the EBQI process involved the localized tailoring of the collaborative-care management program to each CBOC. Researchers ensured that the adaptations were evidence based. Clinical and administrative staff were responsible for adapting the collaborative-care management program for local needs, priorities, preferences and resources. Plan-Do-Study-Act cycles were used to refine the program over time. The evaluation was based on the RE-AIM (Reach, Effectiveness, Adoption, Implementation, Maintenance) Framework and used data from multiple sources: administrative records, web-based decision-support systems, surveys, and key-informant interviews.

**Results:**

*Adoption: *69.0% (58/84) of primary care providers referred patients to the program. *Reach: *9.0% (298/3,296) of primary care patients diagnosed with depression who were not already receiving specialty care were enrolled in the program. *Fidelity: *During baseline care manager encounters, education/activation was provided to 100% (298/298) of patients, barriers were assessed and addressed for 100% (298/298) of patients, and depression severity was monitored for 100% (298/298) of patients. Less than half (42.5%, 681/1603) of follow-up encounters during the acute stage were completed within the timeframe specified. During the acute phase of treatment for all trials, the Patient Health Questionnaire (PHQ9) symptom-monitoring tool was used at 100% (681/681) of completed follow-up encounters, and self-management goals were discussed during 15.3% (104/681) of completed follow-up encounters. During the acute phase of treatment for pharmacotherapy and combination trials, medication adherence was assessed at 99.1% (575/580) of completed follow-up encounters, and side effects were assessed at 92.4% (536/580) of completed follow-up encounters. During the acute phase of treatment for psychotherapy and combination trials, counseling session adherence was assessed at 83.3% (239/287) of completed follow-up encounters. *Effectiveness: *18.8% (56/298) of enrolled patients remitted (symptom free) and another 22.1% (66/298) responded to treatment (50% reduction in symptom severity). *Maintenance: *91.9% (10/11) of the CBOCs chose to sustain the program after research funds were withdrawn.

**Conclusions:**

Provider adoption was good, although reach into the target population was relatively low. Fidelity and maintenance were excellent, and clinical outcomes were comparable to those in randomized controlled trials. Despite the organizational barriers, these findings suggest that EBQI is an effective facilitation strategy for CBOCs.

**Trial registration:**

Clinical trial # NCT00317018.

## Introduction

Collaborative-care management (CCM) is an evidence-based practice that involves a multidisciplinary depression care team (*e.g*., primary care providers, nurse care managers, pharmacists, psychologists, psychiatrists) providing guideline-concordant depression treatment in the primary care setting. Numerous effectiveness studies have demonstrated that CCM improves outcomes for primary care patients treated for depression [1-10]. The CCM model has been rolled out nationally in the United States by the Department of Veterans Affairs (VA) Medical Centers as part of the Primary Care/Mental Health Integration Initiative [11].

More recently, the VA has encouraged the implementation of CCM in its Community Based Outpatient Clinics (CBOCs), where 64% of veterans receive their care [12], and mandated implementation in those categorized as large (5,000-10,000 patients) and very large (> 10,000 patients). Veterans treated at CBOCs have similar demographic characteristics as veterans treated at VA Medical Centers (VAMCs) [13]. All CBOCs provide primary care services, and most large and very large CBOCs also provide specialty mental health services. However, veterans treated in CBOCs have significantly fewer mental health visits than do veterans treated at VAMCs [14]. Twenty-six percent of CBOCs are private clinical organizations contracting with the VA to provide primary care services to veterans [12]. Veterans treated in contract CBOCs have significantly fewer mental health visits than do veterans treated in VA-staffed (*i.e*., owned and operated) CBOCs [15]. While CCM could potentially address this disparity, there are numerous barriers to implementing a complex clinical program like CCM in small contract CBOCs.

The organizational characteristics of contract CBOCs present added challenges to the implementation of CCM. For example, contract CBOCs receive capitated payments (a fixed amount per enrollee to cover a defined scope of services) from the VA and, thus, must consider the financial risk associated with depression quality-improvement efforts. As a result, contract CBOCs may be less willing to comply with VA quality-improvement initiatives compared to VA-staffed clinics, unless these initiatives are embedded into their legal contracts. In addition, the majority of contract CBOCs do not have on-site psychiatrists, and because half of contract CBOCs are located in rural areas, [16] recruiting psychiatrists to small contract CBOCs is typically not feasible. A previous randomized trial documented that the CCM model can be adapted using telemedicine technologies to effectively improve outcomes for patients treated in CBOCs without on-site psychiatrists [17]. While there is good evidence that telemedicine-based CCM improves outcomes in contract CBOCs, no implementation strategy is known to be effective for this type of organizational context.

The overall goal of our research was to facilitate the adoption of telemedicine-based CCM in contract CBOCs. The Promoting Action on Research in Health Services (PARiHS) framework proposes that successful adoption of an evidence-based practice depends on (1) evidence, (2) context, and (3) facilitation [18]. Evidence includes results from randomized trials, as well as anecdotal evidence from clinical experience [19,20]. Context includes both factors internal to the organization, such as culture, climate, and capacity, [21-29] as well as external forces, such as mandates and performance measures. Facilitation typically involves an integrated set of implementation strategies to promote adoption. In this study, we used a facilitation method known as evidence-based quality improvement (EBQI). EBQI has been used successfully to implement CCM in VA Medical Centers [30]. Our specific objective was to test the feasibility of EBQI as an implementation strategy for telemedicine-based CCM in contract CBOCs. Results should inform efforts to roll out complex evidence-based practices to small satellite clinics of integrated healthcare systems.

EBQI was developed by Rubenstein and colleagues based on the findings of the Mental Health Awareness Project, which compared two quality-improvement strategies for depression in primary care [31,32]. Clinics were randomized to either a top-down centralized quality-improvement model or a bottom-up locally driven quality-improvement model [32] The top-down approach involved centralized experts implementing depression evidence-based practices, with some input from local primary care staff. The bottom-up approach involved local clinical staff implementing depression evidence-based practices, with some input from experts. The bottom-up quality-improvement teams had both the best and worst outcomes in terms of fidelity to the evidence base [32]. This finding suggests that the bottom-up approach has the best potential for quality improvement but is subject to substantial variation depending on local climate, culture, and capacity [32]. These findings are consistent with two well-designed implementation studies that found that traditional continuous quality-improvement models do not improve depression outcomes [33,34]. Qualitative analyses of the Mental Health Awareness Project also indicated that the top-down approach was more efficient, but the project failed to attain buy-in from local clinicians and administrators [35]. In contrast, the bottom-up quality-improvement approach promoted customization and buy-in but was perceived to be overly time-consuming and inefficient (*e.g*., reinventing the wheel) [35]. Based on these findings, the EBQI model was developed, which involves both centralized *strategic *decision making and local *tactical *decision making [35]. There is a growing consensus among implementation experts [36-38] and frontline clinicians and managers [32,35,39] that quality-improvement strategies that incorporate both top-down and bottom-up approaches hold the most promise for sustained implementation of evidence-based practices.

In EBQI, both researchers (clinical experts, implementation experts) and local staff participate fully in the quality-improvement process, with the researchers facilitating rather than dictating implementation efforts [32,35,39]. Thus, EBQI is intended to foster a researcher/clinician partnership that promotes buy-in from leadership [40,41]. Lack of support from leadership has been shown to be one of the most important barriers to the implementation of the CCM [42]. While emphasizing the involvement of outside experts and empirical evidence, EBQI stresses that an organization's own healthcare professionals and staff are best positioned to improve their systems [40]. Clinicians and administrators contribute the local knowledge needed to tailor the evidence-based practice for their own particular needs and organizational capabilities. Researchers contribute knowledge of the evidence base; ensure fidelity to the evidence base; and supply materials, procedures, and tools needed for successful implementation. In addition to providing expertise, researchers in the EBQI model also facilitate problem solving and provide ongoing technical support for developing data collection/analysis tools, informatics, and training materials. EBQI also emphasizes continuously revising the adapted evidence-based practice based on feedback during Plan-Do-Study-Act cycles and, thus, should lead to adapted evidence-based practices that are robust, user friendly, and feasible to deploy in real-world practice settings. The primary objective of this research was to test the feasibility of EBQI as a facilitation strategy for implementing telemedicine-based CCM in contract CBOCs.

## Methods

This nonrandomized, small-scale, multisite evaluation of EBQI was conducted at three VAMCs and their affiliated CBOCs located in two regional Veterans Integrated Service Networks (VISNs) in the United States. The VAMCs participating in the implementation were chosen based on their number of affiliated contract CBOCs, their willingness to participate, and their potential for success as perceived by VISN leadership. The three VAMCs were affiliated with 11 contract CBOCs.

### Implementation strategy

Each of the three VAMCs had an EBQI team comprised of stakeholders from mental health, primary care, and the CBOCs, as well as the principal investigator. There were no EBQI meetings involving team members from multiple sites, although the principal investigator shared solutions and tools across sites to increase implementation efficiency. Two of the VAMCs chose to have the telemedicine-based CCM program operated by (*i.e*., administratively housed in) Mental Health, and the third chose to have the program operated by Primary Care. Especially important for this EBQI process was the involvement and buy-in from leadership. Conceptual models of implementation and theories of organizational change strongly emphasize the importance of buy-in from leadership [20,36,43]. Therefore, we designed our EBQI process to engage and leverage clinic leadership. However, personnel from the contract CBOCs could not participate in the EBQI process directly because contract CBOCs were not under the purview of an Institutional Review Board and, therefore, could not be directly engaged in research. Instead, the CBOC liaison at the VAMC represented the view of CBOC stakeholders.

The initial Plan phase of the EBQI process involved the localized tailoring of the telemedicine-based CCM model to each VAMC and their CBOCs. Planning was based on the *Steps and Decisions Guide for Implementing Depression CCM Models*, which was developed specifically for the study [44]. The CCM experts utilized their experience implementing depression research protocols and their knowledge of the depression and quality-improvement literature to ensure the adaptations to the telemedicine-based CCM model were evidence based. The chiefs of mental health/primary care, depression nurse care managers, other clinical leaders at the VAMC, and the CBOC liaisons were responsible for championing the implementation of the program and adapting the telemedicine-based CCM for local needs, priorities, preferences, and resources (within the fidelity parameters defined by the CCM experts). For example, each EBQI team developed unique criteria and methods for identifying which patients were ineligible due to substance dependence. In addition, particular attention was devoted to aligning the telemedicine-based CCM program with the VA depression performance measures. Performance measures are reported by the VA Central Office on a quarterly basis and determine the salary bonuses of VAMC Directors.

The Do phase at each of the VAMCs involved the initial launch of the program, following a site visit from the depression care manager at the CBOC considered to have the highest chances for success. Beginning with just one site allowed us to identify and resolve any unforeseen problems before launching the program at other CBOCs. It also allowed us to share any short-term success experienced by this CBOC with the clinicians at other CBOCs in order to promote provider adoption. Subsequent Do phases involved the launches of the program at the other CBOCs. The Study phase included monthly EBQI conference calls with each site that discussed the implementation efforts. These calls were informed by the data from the Net Decision Support System (NetDSS) depression care manager workload reports that provided information about patient enrollment and fidelity to the care manager protocol (see description below), as well as anecdotal experiences of the care managers and their psychiatrist supervisors. Any implementation problems were discussed during the monthly conference calls and/or via email with the stakeholders. The Act phases of the EBQI process involved the refinement of the adapted telemedicine-based CCM program based on the implementation experiences and data collection described above as the Plan-Do-Study-Act cycle was repeated.

### Training and decision-support tools

The implementation experts also developed training and decision-support tools to promote fidelity to the evidence base. Depression care managers were trained using the *VA Mental Health QUERI Depression Care Manager Training Manual*, which was specifically developed for the study. A PowerPoint (Microsoft Corporation, Redmond, WA, USA) slide set was also developed to facilitate face-to-face training of the depression care managers. In addition, a web-based decision-support system (NetDSS) and a *NetDSS User's Guide *were specifically developed for the study to promote care manager fidelity [45]. Difficulty with information technology is one of the most commonly reported barriers to CCM adoption, [42] especially information technology supporting symptom monitoring [46]. NetDSS is based on a highly structured intervention protocol used for a previous effectiveness study of telemedicine-based CCM [19]. NetDSS provides context-specific decision support in real time during patient encounters by guiding care managers through an evidence-based encounter using self-scoring instruments, scripts, and clinical algorithms to identify new medication trials or counseling trials, treatment phases, and outcome milestones, such as treatment response (*i.e*., 50% reduction in symptom severity), remission (*i.e*., symptom free), and relapse. NetDSS is self-documenting and automatically generates a progress note at the end of the encounter, which was copied and pasted into the electronic health record. NetDSS functionality is divided into five categories: (1) panel management, (2) trial management, (3) clinical decision support, (4) progress note generator, and (5) workload/outcomes report generator. NetDSS was continuously revised based on feedback from the depression nurse care managers as part of the Plan-Do-Study-Act process. The NetDSS workload reports were used to provide fidelity and outcomes data to the EBQI teams for the Plan-Do-Study-Act process.

### Evaluation

The evaluation was based on the RE-AIM (Reach, Effectiveness, Adoption, Implementation, Maintenance) Framework [47-50]. *Reach *represents the absolute number/proportion of eligible/targeted patients who receive the evidence-based practice [47]. *Adoption *represents the absolute number/proportion of staff who use the evidence-based practice [47]. *Implementation *represents the fidelity of the evidence-based practice as implemented in routine care [47]. *Effectiveness *represents the clinical impact (on patient outcomes) of the evidence-based practice as implemented in routine care settings [49]. *Maintenance *represents the degree to which the implementation of the evidence-based practice is sustained [47]. To have an impact on health at the population level, an intervention must be *adopted *by providers, *reach *a large proportion of the targeted patient population, be *implemented *with fidelity, *effectively *improve outcomes, and be *maintained *after research funds are withdrawn. In addition, we measured implementation costs.

### Adoption

To measure adoption, data were extracted from the Medical SAS Datasets at the Austin Information Technology Center. The post-period was defined as the 12 months after each CBOC start date (defined as the date the first patient was enrolled in the telemedicine-based CCM program), which ranged from April 2006 to February 2008. The number of primary care providers at each CBOC was determined from the SAS Medical Datasets using unique provider IDs for all types of primary care providers (*e.g*., general internist, advance practice nurse). The number of primary care providers referring a patient to a depression care manager during the 12-month post-period was identified from NetDSS. The adoption rate for each CBOC was defined as the total number of primary care providers referring a patient to the depression care manager (identified from NetDSS) during the 12-month period divided by the total number of primary care providers seeing patients during the 12-month period (identified from SAS Medical Datasets).

### Reach

To measure reach, data were extracted from the SAS Medical Datasets at the Austin Information Technology Center for the 12-month post-period. Index visits for patients during the 12-month post-period were defined as the first primary care encounter at the CBOC with a depression diagnosis. Patients were excluded if they had a specialty mental health visit or a diagnosis of bipolar disorder or schizophrenia during the six months prior to the CBOC start date or if the index visit was a specialty mental health encounter. The number of patients referred to the depression care manager during the 12-month post-period was identified from NetDSS. Reach during the 12-month post-period at each CBOC was defined as the total number of patients referred to the depression care manager (identified from NetDSS) divided by the total number of patients with a depression diagnosis who were not already receiving specialty care (identified in the SAS Medical Datasets).

### Implementation/fidelity

To measure fidelity, data were extracted from NetDSS for the 12-month post-period. NetDSS automatically collects data about whether care-manager modules were completed during care-manager encounters. Fidelity during the acute and continuation phases of treatment was reported in aggregate using the NetDSS outcomes reports routine. For the first encounter during the acute phase of treatment, the fidelity measures included the proportion of patients receiving education/activation, barrier assessment/resolution, and Patient Health Questionnaire (PHQ9) depression symptom monitoring. For follow-up encounters during the acute phase of treatment, fidelity measures included the proportion of encounters where self-management goals were discussed, depression symptoms were monitored, medication adherence and side-effects were assessed (for pharmacotherapy and combination pharmacotherapy/psychotherapy trials only), and counseling adherence was assessed (for psychotherapy and combination trials only). In addition, NetDSS reports what portion of follow-up encounters were completed within the prespecified timeframe: every two weeks in the acute phase for pharmacotherapy and combination pharmacotherapy/psychotherapy trials, every four weeks for watchful waiting and psychotherapy-only trials, and every four weeks for all trials in the continuation phase.

### Effectiveness

To measure effectiveness, data were extracted from the NetDSS final disposition codes for patients enrolled during the 12-month post-period. Final disposition codes were as follows: 1 = remitted and completed the continuation, 2 = responded (> 50% reduction in PHQ9 score) and completed the continuation phase without relapsing, 3 = unable to complete baseline assessment or lost to follow-up, 4 = disenrolled at patient's request, 5 = disenrolled at provider's request, 6 = became ineligible (*e.g*., entered a nursing home or moved away), and 7 = referred to a higher level of care (*e.g*., specialty mental health) due to lack of response or detection of complex psychiatric comorbidities.

### Maintenance

Maintenance represents the sustainability of the telemedicine-based CCM program and the extent to which it has been institutionalized into the organization's practices and policies. Maintenance was measured using two complementary instruments approximately a year after the last CBOC enrolled its first patient into the telemedicine-based CCM program, when research funds were no longer supporting the salary of clinical personnel. The Level of Use interview [51] measured sustained use of the program, and the Level of Institutionalization survey [52] measured the degree to which the program was institutionalized within the organization. Level of Use was measured using key informant interviews with the Medical Directors of each of the 11 CBOCs and the Chief of Mental Health or Chief of Primary Care at the VAMC (depending on which service line operated the program). Using a structured interview guide and inductive questioning, the Level of Use framework classified the CBOCs into eight ranked levels of adoption according to their adoption behaviors. The first three levels distinguish between stages of nonuse (nonuse, orientation, and preparation). The next five levels distinguish between stages of use (mechanical, routine, refinement, integration, renewal), and these distinctions are made based on the type of adaptations or refinements that are being made to the innovation. Level of Institutionalization was measured via telephone survey of the Chief of Mental Health or Chief of Primary Care at the VAMC (depending on which service line operated the program). Institutionalization implies that the organization has modified itself to incorporate the innovation and that the innovation has ceased to become novel and has been embedded in standard operating procedures. The Level of Institutionalization instrument measures an innovation's institutionalization among four subsystems: production, maintenance, supportive, and managerial. The production subsystem is responsible for delivering clinical services; to be institutionalized, the innovation must be integrated with other routine clinical services. The maintenance subsystem represents personnel; to be institutionalized, the innovation must be supported by permanent employees. The supportive subsystem represents external organizational forces; to be institutionalized, the innovation must have a stable source of funding and permanent office space. The managerial subsystem represents the executive and supervisory functions; to be institutionalized, the innovation must be assigned to a specific service, staff must have written job descriptions, and performance measures and progress reports must be required. For each subsystem, the Level of Institutionalization survey asks the respondent about the degree to which the organization has institutionalized the innovation (*e.g*., supported by permanent employees), and the responses are averaged to calculate an overall mean for each subsystem. The Level of Institutionalization instrument has three levels for each subsystem: low institutionalization (mean score ≤ 2), moderate institutionalization (2 < mean score ≤ 3) and high institutionalization (mean score > 3).

### Pre-implementation planning and fixed implementation costs

Pre-implementation planning costs represent the cost that a VAMC would incur to implement a telemedicine-based CCM model in CBOCs. The total cost of attending EBQI meetings was calculated by multiplying the number of meetings each EBQI member attended by their hourly wage estimated from their grade and step (or nurse level). The total cost of implementation activities between EBQI meetings was calculated by multiplying the estimated hours per month devoted to implementation activities by the hourly wage estimated from grade and step (or nurse level). Fixed implementation costs represent the one-time cost of implementing telemedicine-based CCM, including the development of the *VA Mental Health QUERI Depression Care Manager Training Manual*, NetDSS (web-based decision support system), and the *NetDSS User's Guide*. These costs were calculated by estimating the number of hours worked on each of these activities and from final budget expenditures. These fixed costs would not be incurred for VAMCs implementing the telemedicine-based CCM model in CBOCs in the future.

The research was approved by the Institutional Review Boards of the Central Arkansas Veterans Healthcare System, the Greater Los Angeles Healthcare System, and the Loma Linda Healthcare System.

## Results

### Characteristics of contract community based outpatient clinics

The characteristics of the 11 participating CBOCs are presented in Table 1. During the 12-month post-period, 42,330 unique patients were treated at the 11 CBOCs. The number of patients ranged from 1,325 to 7,411 across the CBOCs, placing the clinics in the small (< 1,500 patients), medium (1,500-5,000 patients), and large (5,000-10,000 patients) categories. On average, the percent of patients diagnosed with depression (but without a specialty mental health visit or a diagnosis of bipolar disorder or schizophrenia) ranged from 4.5% to 12.3%, with an average of 7.8% across the CBOCs. Among this group of patients eligible for depression care management (n = 3,296), the number of depression-related primary care encounters per patient during the 12-month post-period ranged from 1.1 to 1.8, with an average of 1.3 (standard deviation [SD] = 0.6), which was about two-thirds of the total number of primary care encounters. Also among this group, the number of depression-related specialty mental health visits during the 12-month post-period ranged from 0.3 to 1.4, with an average of 0.5 (SD = 1.8), which accounted for about half of the total number of specialty mental health visits.

**Table 1:** Patterns of depression treatment during the 12-month post-implementation period by study site

Facility	Unique number of patients	Unique number of patients diagnosed with depression	Mean primary care visits per patient	Mean primary care visits for depression per patient	Mean specialty mental health visits per patient	Mean specialty mental health visits for depression per patient
**VAMC A**						
CBOC 1	3474	243 (7.0%)	2.0	1.2	0.8	0.4
CBOC 2	1968	112 (5.7%)	1.9	1.3	1.2	0.8
CBOC 3	4534	487 (10.7%)	1.7	1.3	0.8	0.3
CBOC 4	1325	163 (12.3%)	2.1	1.8	1.8	1.4
**VAMC B**						
CBOC 5	4545	203 (4.5%)	1.9	1.2	1.6	0.4
CBOC 6	5341	431 (8.1%)	1.7	1.2	1.1	0.6
CBOC 7	7411	673 (9.1%)	1.6	1.3	0.8	0.4
CBOC 8	2374	162 (6.8%)	1.8	1.3	2.0	0.9
CBOC 9	3642	182 (5.0%)	1.8	1.1	0.9	0.5
**VAMC C**						
CBOC 10	3252	279 (8.6%)	2.0	1.3	1.1	0.6
CBOC 11	4464	361 (8.1%)	2.4	1.4	1.0	0.6
Total	42,330	3,296 (7.8%)	1.9 (SD = 1.2)	1.3 (SD = 0.6)	1.1 (SD = 4.7)	0.5 (SD = 1.8)

*VAMC *= Department of Veterans Affairs Medical Center; *CBOC *= Community Based Outpatient Clinic; *SD *= standard deviation.

*VA *= Department of Veterans Affairs; *CBOC *= Community Based Outpatient Clinic.

### Evidence-based quality-improvement process

An average of 11.7 (range = 9-14) staff members participated in at least one EBQI meeting at each of the affiliated VAMCs, but only 4.7 (range = 3-6) participated in five or more EBQI meetings on average. The duration of the EBQI process averaged 21.3 months (range = 16-25) across the three VAMCs, and the average number of EBQI meetings was 13.7 (range = 13-15). Of those participating in five or more meetings, the average number of hours per month spent on implementation efforts was 13.3 (range = 5.5-19.6). Of the EBQI team members, the depression nurse care managers spent the most hours per month on implementation efforts (mean = 28.7, range = 16-45).

### Collaborative-care management program structure

All three VAMCs chose to include one depression nurse care manager available by telephone and one supervising tele-psychiatrist on the CCM team. The two programs that were operated by Mental Health were able to obtain psychiatric supervision for the depression care managers more easily than the site operated by Primary Care. Other types of providers (*e.g*., pharmacists, psychotherapists) were not included on any of the CCM teams. Each VAMC chose to pilot the telemedicine-based CCM program at one CBOC first and then spread the program to other CBOCs affiliated with the VAMC. All three VAMCs chose to enroll only patients who were referred to the care manager by their primary care provider. In contrast, none of the VAMCs chose to target all patients screening positive for depression, or all patients diagnosed with depression, or all patients initiating antidepressant treatment. However, all three VAMCs did choose to use an existing Depression Case Finder to identify patients targeted by VA depression performance measures (*e.g*., those with a new diagnosis and a new antidepressant prescription) and to request consults (*i.e*., provider referrals) to the program for these patients. In addition, care manager clinic codes, diagnoses, and Current Procedural Terminology (CPT) codes were chosen to ensure that care manager encounters contributed to the VA depression follow-up visit performance measure. All three VAMCs chose to exclude patients with serious mental illnesses, as well as patients already receiving (or eventually referred) to specialty mental health. However, in actuality, care managers sometimes continued to provide care management to patients referred to their supervising tele-psychiatrist for ongoing care. Care management activities at all three VAMCs included education/activation, barrier assessment/resolution, symptom monitoring, medication adherence monitoring, side-effects monitoring, and self-management. All of the VAMCs chose to exclude brief counseling as a care manager activity to maximize the caseload capacity of the care manager. None of the VAMCs developed formal guidelines (*e.g*., decision nodes) for referring patients from the telemedicine-based CCM program to specialty mental health (*i.e*., to a higher level of care), but rather, this decision was left to the discretion of the care manager's supervising tele-psychiatrist.

### Adoption

**Figure 1:**
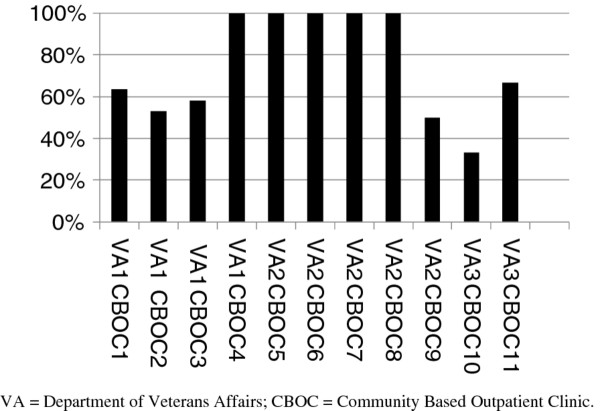
**Percentage of providers referring to the depression care manager**. To measure adoption, data were extracted from the Medical SAS Datasets at the Austin Information Technology Center. The post-period was defined as the 12 months after each CBOC start date (defined as the date the first patient was enrolled in the telemedicine-based CCM program), which ranged from April 2006 to February 2008. The number of primary care providers at each CBOC was determined from the SAS Medical Datasets using unique provider IDs for all types of primary care providers (*e.g*., general internist, advance practice nurse). The number of primary care providers referring a patient to a depression care manager during the 12-month post-period was identified from NetDSS. The adoption rate for each CBOC was defined as the total number of primary care providers referring a patient to the depression care manager (identified from NetDSS) during the 12-month period divided by the total number of primary care providers seeing patients during the 12-month period (identified from SAS Medical Datasets).

There were 84 primary care providers who diagnosed one or more patients with depression at the implementation CBOCs during the 12-month post-period. Approximately 69.0% (58/84) of these providers referred at least one patient to the depression care manager. Adoption rates ranged across CBOCs from a low of 33.3% to a high of 100% (see Figure 1).

### Reach

**Figure 2:**
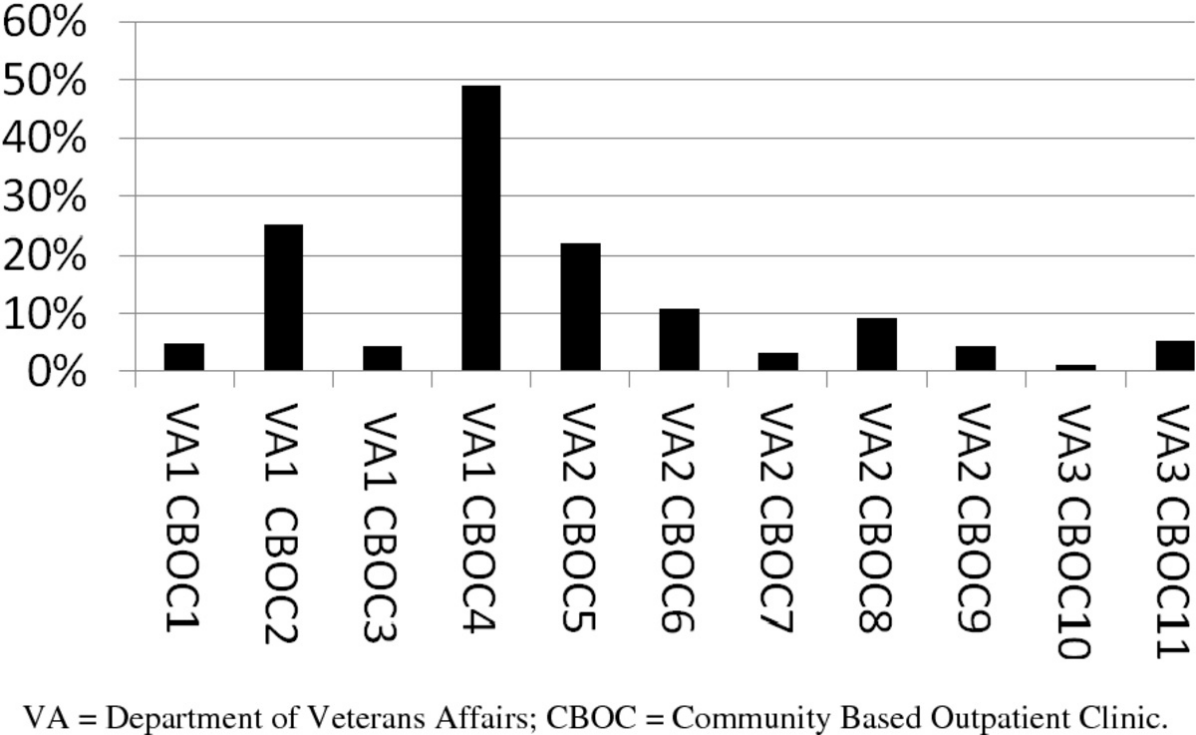
**Percentage of patients referred to the depression care manager**. To measure reach, data were extracted from the SAS Medical Datasets at the Austin Information Technology Center for the 12-month post-period. Index visits for patients during the 12-month post-period were defined as the first primary care encounter at the CBOC with a depression diagnosis. Patients were excluded if they had a specialty mental health visit or a diagnosis of bipolar disorder or schizophrenia during the six months prior to the CBOC start date or if the index visit was a specialty mental health encounter. The number of patients referred to the depression care manager during the 12-month post-period was identified from NetDSS. Reach during the 12-month post-period at each CBOC was defined as the total number of patients referred to the depression care manager (identified from NetDSS) divided by the total number of patients with a depression diagnosis who were not already receiving specialty care (identified in the SAS Medical Datasets).

There were 3,296 patients diagnosed with depression (but without a prior specialty mental health visit or a diagnosis of bipolar disorder or schizophrenia) at implementation CBOCs during the 12-month post-period. Nine percent (298/3296) of these patients had an encounter with a depression care manager. Reach ranged across CBOCs from a low of 1.1% to a high of 49.1% (see Figure 2).

### Implementation/fidelity

For most domains, fidelity to the care manager protocol was excellent for the 298 patients who had encounters with a depression care manager. Patient education/activation and barriers assessment/resolution were provided at 100% (298/298) of baseline encounters (*i.e*., the first care manager encounter). Likewise, the PHQ9 depression symptom-monitoring tool was used to assess depression severity at 100% (298/298) of baseline encounters. The 298 patients had 368 treatment trials in the acute phase. Almost half (48.4%, 178/368) of the acute phase trials were pharmacotherapy only, 28.8% (106/368) were combination pharmacotherapy/psychotherapy, 6.3% (23/368) were psychotherapy only, and 16.6% (61/368) were watchful waiting. Less than half (42.5%, 681/1603) of follow-up encounters during the acute stage were completed within the timeframe specified in the acute phase of treatment (*i.e*., within two or four weeks, depending on the trial type). During the acute phase of treatment for all trials, the PHQ9 symptom-monitoring tool was used at 100% (681/681) of completed follow-up encounters, and self-management goals were discussed during 15.3% (104/681) of completed follow-up encounters. During the acute phase of treatment for pharmacotherapy and combination trials, medication adherence was assessed at 99.1% (575/580) of completed follow-up encounters, and side effects were assessed at 92.4% (536/580) of completed follow-up encounters. During the acute phase of treatment for psychotherapy and combination trials, counseling-session adherence was assessed at 83.3% (239/287) of completed follow-up encounters. During the continuation phase of treatment, 54.6% (189/346) of follow-up encounters were completed within the prespecified timeframe (*i.e*., within four weeks).

### Effectiveness

Depression care managers enrolled 298 patients into the telemedicine-based CCM program. Of these, 7.4% (22/298) could not be reached for a baseline evaluation and another 8.7% (26/298) were lost to follow-up. In addition, 9.7% (29/298) were disenrolled at the patient's request and 0.3% (1/298) were disenrolled at the primary care provider's request. Another 7.7% (23/298) became ineligible (*e.g*., moved out of area), and 24.2% (72/298) were referred to a higher level of care (*e.g*., referred to specialty mental health). Overall, 18.8% (56/298) remitted (symptom free) and completed the continuation phase of treatment and another 22.1% (66/298) responded to treatment and completed the continuation phase without relapsing. Thus, 40.9% (122/298) of enrolled patients had positive outcomes because they either remitted or responded and completed without relapsing. Examining outcomes for completers only (n = 194), 34.0% (66/194) responded to treatment and completed, 28.8% (56/194) remitted and completed (62.8% positive outcomes, 122/194), and 37.1% (72/194) were referred to a higher level of care.

### Maintenance

**Figure 3:**
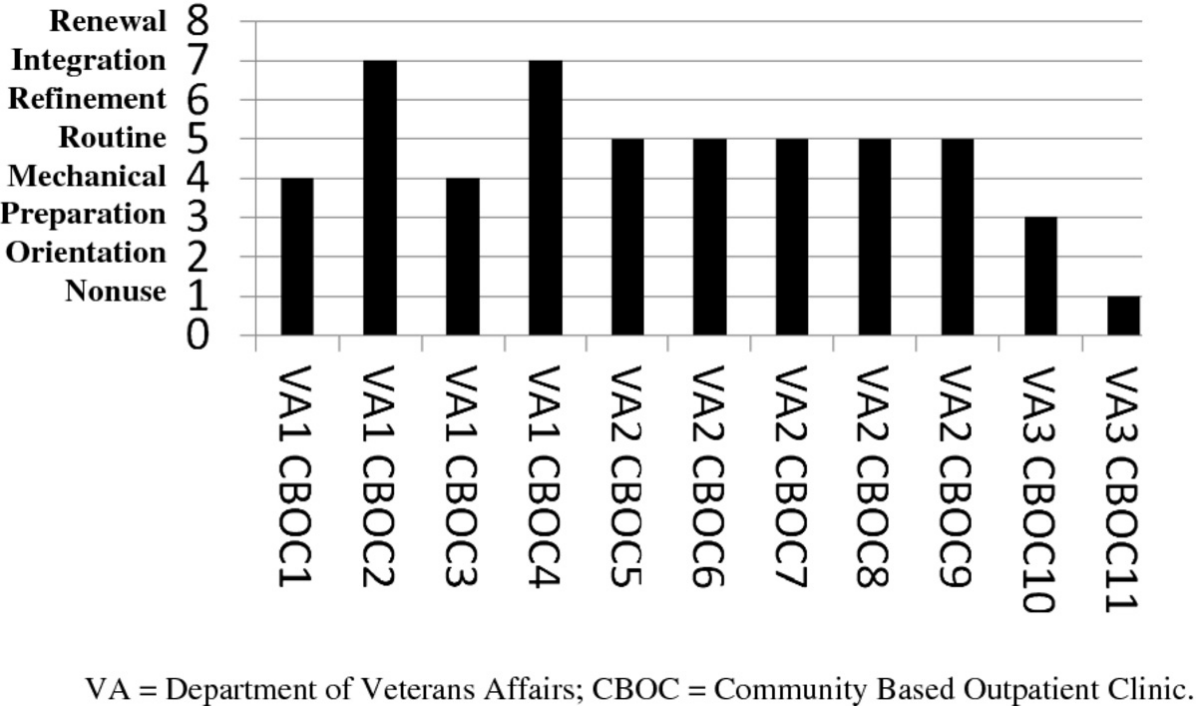
**Level of use of telemedicine-based collaborative-care management**. The Level of Use interview measured sustained use of the program and was administered approximately a year after the last CBOC enrolled its first patient into the telemedicine-based CCM program, when research funds were no longer supporting the salary of clinical personnel. Level of Use was measured using key informant interviews with the Medical Directors of each of the 11 CBOCs and the Chief of Mental Health or Chief of Primary Care at the VAMC (depending on which service line operated the program). Using a structured interview guide and inductive questioning, the Level of Use framework classified the CBOCs into eight ranked levels of adoption according to their adoption behaviors. The first three levels distinguish between stages of nonuse (nonuse, orientation, and preparation). The next five levels distinguish between stages of use (mechanical, routine, refinement, integration, renewal), and these distinctions are made based on the type of adaptations or refinements that are being made to the innovation.

**Figure 4:**
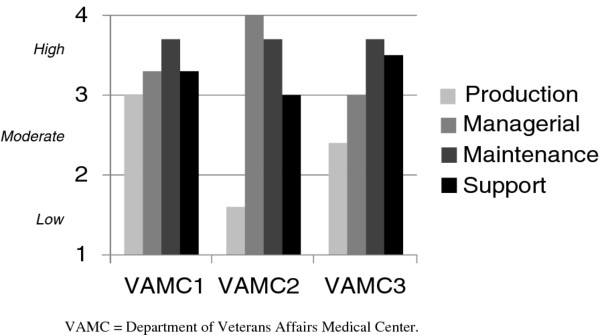
**Level of institutionalization of telemedicine-based collaborative care management**. Level of Institutionalization survey measured the degree to which the program was institutionalized within the organization. Level of Institutionalization was measured via telephone survey of the Chief of Mental Health or Chief of Primary Care at the VAMC (depending on which service line operated the program). Institutionalization implies that the organization has modified itself to incorporate the innovation and that the innovation has ceased to become novel and has been embedded in standard operating procedures. The Level of Institutionalization instrument measures an innovation's institutionalization among four subsystems: production, maintenance, supportive, and managerial. The production subsystem is responsible for delivering clinical services; to be institutionalized, the innovation must be integrated with other routine clinical services. The maintenance subsystem represents personnel; to be institutionalized, the innovation must be supported by permanent employees. The supportive subsystem represents external organizational forces; to be institutionalized, the innovation must have a stable source of funding and permanent office space. The managerial subsystem represents the executive and supervisory functions; to be institutionalized, the innovation must be assigned to a specific service, staff must have written job descriptions, and performance measures and progress reports must be required. For each subsystem, the Level of Institutionalization survey asks the respondent about the degree to which the organization has institutionalized the innovation (*e.g*., supported by permanent employees), and the responses are averaged to calculate an overall mean for each subsystem. The Level of Institutionalization instrument has three levels for each subsystem: low institutionalization (mean score ≤ 2), moderate institutionalization (2 < mean score ≤ 3) and high institutionalization (mean score > 3).

There was variation across the 11 CBOCs on the Level of Use instrument. One (9.1%) CBOC was classified as a nonuser (level 1), two (18.2%) were classified as mechanical users (level 3), one (9.1%) was classified as a refinement user (level 6), and seven (63.6%) were classified as integrated users (level 7) (see Figure 3). Level of Institutionalization was mostly in the high to moderate range across all three VAMCs (see Figure 4).

### Pre-implementation planning costs and fixed costs

The total cost of attending EBQI meetings was $8,270.90. The total costs for the time spent on implementation between EBQI meetings was $84,483.45. The total pre-implementation planning costs across all three VAMCs were estimated to be $92,753.79. Dividing total pre-implementation planning costs by the 3,296 patients diagnosed with depression at implementation CBOCs and targeted by the telemedicine-based CCM program during the 12-month post-period yields a total pre-implementation planning cost per targeted patient of $28.14. Dividing total pre-implementation planning costs by the 298 patients enrolled in the telemedicine-based CCM program during the 12-month post-period yields a total pre-implementation planning cost per patient reached of $311.25. The fixed cost for developing the *Mental Health QUERI Depression Care Manager Training Manual *was $17,182. The fixed cost of developing NetDSS was $100,370. The fixed cost for developing the *NetDSS User's Guide *was $12,391. The total fixed costs were $129,943.

## Discussion

The telemedicine-based CCM program had an excellent adoption rate by primary care providers (69%), although reach into the target patient population was relatively low overall (9%). The low proportion of targeted patients reached by the program is somewhat difficult to interpret, as it is not clear what proportion of patients with depression would have benefited clinically from depression care management. Moreover, because the EBQI teams specifically chose to target those patients whose primary care providers thought would benefit from the telemedicine-based CCM program, it made it impossible to identify in a systematic manner what the true denominator was for the reach evaluation. Presumably, many of the patients diagnosed with depression (used as the dominator for the reach evaluation) were in remission or stable on medications during the time period and would not have benefited clinically from the program. Of those enrolled in the telemedicine-based CCM program, fidelity to the care manager protocol was excellent. The high level of fidelity can be attributed, in large part, to the use of the NetDSS care manager decision support system. NetDSS workload/outcomes reports were discussed at EBQI team meetings and used to address problems with care manager fidelity. The biggest problems with fidelity were completing assessments within the prespecified timeframe and care manager activities that were not required to be completed by NetDSS (*i.e*., the optional self-management planning). In addition, the clinical outcomes of enrolled patients were comparable to intervention patients in a prior randomized effectiveness study of telemedicine-based CCM [19]. Of those enrolled, 18.8% completed the continuation phase in remission and another 22.1% responded to treatment and completed the continuation phase without relapsing. Thus, from an intent-to-treat perspective, 40.9% of patients enrolled in telemedicine-based CCM had positive outcomes, which is somewhat higher than the 12-month intent-to-treat response rate (36%) reported in a previous randomized effectiveness trial of telemedicine-based CCM that enrolled patients screening positive for depression [19]. A previous study has demonstrated that patients referred to CCM by their primary care provider as part of routine care experience greater symptom improvement than those randomized to CCM after being enrolled in a research study using traditional recruitment methods [53]. For those completing the telemedicine-based CCM program, 65.8% either remitted or responded without relapsing. Importantly, the telemedicine-based CCM programs continued to be used in a sustained manner after research funds were withdrawn. Likewise, the telemedicine-based CCM programs became institutionalized into the operations of the affiliated VAMCs.

Based on these findings, we conclude that EBQI is a feasible facilitation strategy for contract CBOCs. This finding is significant because there were many barriers to adoption in contract CBOCs, and these results are a testament to the strength of this particular facilitation method. Rogers has argued that innovation complexity is a barrier to diffusion, [54,55] and telemedicine-based CCM is a multifaceted intervention that involves a multidisciplinary care team and, thus, is relatively complex to put into practice. Rogers also argued that incompatibility can be a barrier to adoption, [54,55] and telemedicine-based CCM was not compatible with traditional referral-based treatment patterns of depression treatment in the VA. Because the VA is an integrated system of care, referrals from primary care to specialty mental health care are common, even for mild to moderate depression. Because CCM encourages a more integrated approach to care, it was somewhat disruptive to put into practice. However, there was anecdotal evidence that implementing telemedicine-based CCM in CBOCs was less disruptive than implementing practice-based CCM in parent VAMCs. This may have been due to the long travel distance to specialty mental healthcare for CBOC patients, which may have resulted in the CBOC providers being less reliant on referrals than primary care providers at parent VAMCs. The EBQI process also overcame other barriers to diffusion identified by Rogers, especially trialability [54,55]. Trialability is the degree to which an innovation can be tested on a limited basis and was a barrier to the adoption because patients can remain in care management for 6 to 12 months. Piloting telemedicine-based CCM in the Do phase facilitated the ability to "test drive" the intervention.

As discussed in the PARiHS framework, context is also an important factor to the adoption of evidence-based practices [20]. With respect to external contextual factors, the sustained implementation of telemedicine-based CCM was facilitated by the VA's national Primary Care/Mental Health Integration Initiative, which concurrently promoted the implementation of CCM models. Even though CCM implementation was only mandated for the one large CBOC in our sample, the implementation of the telemedicine-based CCM program was highly compatible [54,55] with the existing priorities of the VA Central Office. In contrast to the favorable external context of implementing telemedicine-based CCM, the internal context of contract CBOCs was a barrier to implementation. Contract CBOCs are likely to have very different organizational cultures and climates than VAMCs and VA-staffed CBOCs. Likewise, because contract CBOCs receive capitated payments, they have a financial incentive to minimize costs, including both patient care expenses and the investment of slack resources in quality-improvement efforts. Although empirical evidence indicates that telemedicine-based CCM does not increase primary care visits or costs [54], the additional cost of collaborating clinically with an off-site care team may have been a concern of the primary care providers. This concern may have led to fewer referrals, thereby contributing to the low level of reach into the target population.

It is also important to note that the EBQI implementation strategy was relatively resource intensive. Ignoring the fixed cost of developing the training materials and decision support system, the pre-implementation planning cost per patient reached was $311. It required the commitment of a large number of busy administrators and clinicians, as well as the involvement of implementation researchers. Therefore, the EBQI implementation strategy used in this study is probably not particularly scalable. However, assuming that the sites included in this study are generalizable to other CBOCs, it is probably not necessary to repeat our relatively resource-intensive adaption process to spread the telemedicine-based CCM to other sites [55]. Presumably, much of the spread to other CBOCs could be more standardized than it was in this study. A more scalable (*i.e*., less intensive) EBQI strategy could also be employed to support spread, perhaps involving larger groups of CBOCs in the Plan-Do-Study-Act cycles.

An important limitation of this study is that VAMCs were chosen based on their willingness to participate and their potential for success as perceived by VISN leadership. While the results from these implementation sites may not be generalizable to all VAMCs, it is advisable to initially choose sites that are committed to implementation for small-scale, multisite evaluation studies. Similar to an efficacy study, the purpose of small-scale, multisite evaluation is to determine whether implementation strategies can work under favorable circumstances [56]. Generalizability of sites becomes a more critical issue for region-wide demonstration studies, where the purpose is to determine whether promising implementation strategies are effective under a wide range of contexts [56]. A second limitation was that we did not have the resources to collect qualitative data about provider adoption and patient reach. Qualitative data obtained from key informant interviews with frontline providers would have helped us better interpret our quantitative findings about adoption and reach. Another limitation of the study was that, due to lack of Institutional Review Board oversight at contract CBOCs, on-site providers and administrators could not be engaged in research and, therefore, could not participate directly in the EBQI process. This was an artificial barrier to implementation that we encountered in the context of conducting research that would not be an issue outside this context. Finally, this feasibility study had no comparison group that used an alternative implementation strategy, and thus, we only demonstrated that EBQI can be successful in this context, not that it was more effective than other implementation strategies.

The major strength of the evaluation component of the study was the integration of clinical information obtained from administrative data and the decision support system (NetDSS) to measure adoption, reach, implementation/fidelity, and effectiveness. Likewise, another strength was measuring maintenance by integrating quantitative and qualitative data obtained from the Level of Institutionalization survey and the Level of Use structured key-informant interviews. The major strength of the implementation component of the study was development and refinement of the decision support system (NetDSS), which substantially enhanced care manager fidelity. The use of NetDSS has spread to non-study sites at one of the regional VISNs and is now being used for other mental health disorders. Based on the results of a previous effectiveness study and the implementation tools developed for this study, telemedicine-based CCM is now listed on the National Registry of Evidence-based Programs and Practices (NREPP), a service of the Substance Abuse and Mental Health Services Administration (SAMHSA). NREPP is a searchable database of interventions, and SAMHSA has developed this resource to help agencies and organizations implement evidence-based mental health practices in their communities.

## Conclusions

The results of this study suggest that EBQI can be used successfully to implement a complex disruptive intervention (telemedicine-based CCM) with fidelity, despite the organizational barriers in contract CBOCs. The success of the EBQI process was likely due to the partnership between clinical leaders and researchers. The researchers were able to develop evidence-based training materials and decision-support tools that seemed to promote fidelity and effectiveness. In turn, the clinical leaders were able to align the telemedicine-based CCM program with the needs of CBOC staff and patients, which seemed to promote adoption, reach, and maintenance. Given that EBQI is resource intensive, it may be a particularly appropriate facilitation strategy during the early stages of an implementation initiative when a relatively small number of sites are committed to successful implementation.

## Competing interests

The authors declare that they have no competing interests.

## Authors' contributions

JF obtained funding and contributed to study conceptualization and study design, acquisition of data, statistical analysis, interpretation of data, and drafting of the manuscript. ME and SM obtained funding, contributed to study conceptualization and study design, and critically revised the manuscript for important intellectual content. JC, JO, and LA supervised the clinical team, provided administrative leadership, and critically revised the manuscript for important intellectual content. GC helped with study conceptualization and study design, collection of Level of Use data from key-informant interviews, interpretation of data, drafting of manuscript, and critical revision of the manuscript for important intellectual content. All authors read and approved the final manuscript.
